# Proteomic Profiling and Monitoring of Training Distress and Illness in University Swimmers During a 25-Week Competitive Season

**DOI:** 10.3389/fphys.2020.00373

**Published:** 2020-05-25

**Authors:** Amy M. Knab, David C. Nieman, Laura M. Zingaretti, Arnoud J. Groen, Artyom Pugachev

**Affiliations:** ^1^Department of Kinesiology, Queens University of Charlotte, Charlotte, NC, United States; ^2^North Carolina Research Campus, Appalachian State University, Kannapolis, NC, United States; ^3^Centre for Research in Agricultural Genomics, Barcelona, Spain; ^4^ProteiQ Biosciences GmbH, Potsdam, Germany

**Keywords:** swimming, proteins, inflammation, upper respiratory tract infection, mental stress

## Abstract

**Purpose:**

To evaluate relationships of proteomics data, athlete-reported illness, athlete training distress (TDS), and coaches’ ratings of distress and performance over the course of the competitive season.

**Methods:**

Thirty-five NCAA Division II swimmers were recruited to the study (male *n* = 19, female *n* = 16; age 19.1 ± 1.6 years). Athletes provided fingerprick dried blood spot (DBS) samples, illness symptoms, and TDS every Monday for 19 of 25 weeks in their season. Coaches monitored performance and rated visual signs of distress. DBS samples were analyzed for a targeted panel of 12 immune-related proteins using liquid chromatography/mass spectrometry (LC/MS).

**Results:**

Thirty-two swimmers completed the protocol. The data were grouped in 2–3 weeks segments to facilitate interpretation and analysis of the data. TDS scores varied between athletes, and were highest during the early fall conditioning ramp up period (8.9 ± 1.6 at baseline to a peak of 22.6 ± 2.0). The percent of athletes reporting illness was high throughout the season (50–78%). Analysis of TDS using Principle Component Analysis (PCA) revealed that 40.5% of the variance (PC1) could be attributed to illness prevalence, and TDS scores for the athletes reporting illness and no illness were different across the season (*P* < 0.001). The coaches’ ratings of swim performance and swimmer’s distress, sex, and racing distance (sprinters, middle distance, long distance) were not correlated with PC1. Linear Discriminant Analysis (LDA) analysis of the data showed a separation of the baseline weeks from exam weeks with or without competitions, and with competitions alone (*p* < 0.001). Seven of the 12 proteins monitored over the course of training were upregulated, and the addition of the protein data to LDA analysis enhanced the separation between these groups of weeks.

**Conclusion:**

TDS and illness were related in this group of 32 collegiate swimmers throughout the competitive season, and expression of immune proteins improved the statistical separation of baseline weeks from the most stressful weeks. TDS data provided by the swimmers did not match their coaches’ ratings of distress and swim performance. The importance of the immune system in the reaction to internal and external stress in athletes should be an area of further research.

## Introduction

The linkage between intensive exercise training and increased risk for illness has been an active area of research during the past several decades. Studies indicate that illness risk may be elevated when an athlete engages in recurring cycles of unusually intense exercise training coupled with competitive events (Nieman, 2000; Nieman and Wentz, 2019). In addition, periods of overreaching for any athlete, although necessary for further adaptations in performance, come with significant physiologic changes that may compromise the immune system. Swimmers are an interesting cohort of athletes that face many barriers to performance including balancing training with outside stressors.

High-level university swimmers for example, participate in 10–15 competitions during the fall and winter seasons when pathogen exposure is high, train 3–5 h daily, experience heavy travel schedules that disrupt sleep and eating habits, and undergo high mental stress from educational and personal commitments (Knab et al., 2013; Hellard et al., 2015; Collette et al., 2018). As a result, illness risk can be elevated, interfering with the capacity to train and race (Gleeson et al., 1995, 2000; Fricker et al., 2005). A 4-year study of 28 elite swimmers showed that the odds for upper respiratory tract infection (URTI) increased 1.08 and 1.10 times for every 10% increase in resistance and high-load training, respectively (Hellard et al., 2015).

In response, several athletic organizations including the International Olympic Committee (IOC) initiated acute illness surveillance systems to delineate the extent of the problem and underlying risk factors (Mountjoy et al., 2010, 2015; Schwellnus et al., 2016; Soligard et al., 2016, 2017; Drew et al., 2017, 2018; Prien et al., 2017). The IOC has focused on inappropriate management of both internal (e.g., mental stress responses) and external loads (e.g., exercise training and competition workloads) (Schwellnus et al., 2016; Soligard et al., 2017). Load management is a key strategy, according to the IOC, to reduce illness incidence and associated downturns in exercise performance, interruptions in training, missed competitive events, and risk of serious medical complications. The IOC recommends monitoring for early signs and symptoms of over-reaching, overtraining, and illness, and to periodically assess psychological stresses using available instruments.

Multiple studies have established that a mismatch exists between the exertion stress perceived by athletes and that intended by coaches (Brink et al., 2014, 2017). For this and other reasons, there is growing interest in the application of practical and efficacious blood and urine measures that can be used to improve the detection of overreaching and overtraining in athletes. Unfortunately, reliable biomarkers that are sensitive to the training load and occur prior to the establishment of overreaching, training distress, and illness have not yet been identified (Meeusen et al., 2013). Subjective measures (i.e., survey data, rate perceived exertion, and Training Distress Scale) are currently regarded as superior to physiological measures such as plasma hormones and cytokines, energy homeostasis, and exercise workload monitoring (Jürimäe et al., 2011; Saw et al., 2016; Joro et al., 2017).

Current advancements in the field of proteomics may provide a mechanism to identify target biomarkers for overreaching in athletes. Proteomics involves the large scale measurement of the structure and function of proteins in a tissue or organism, and is useful in the identification of candidate biomarkers for various disease processes and drug treatments (Rodríguez-Suárez and Whetton, 2013). Even though proteins are the main components of the metabolic pathways of cells, proteomics, until recently, has seldom been used in exercise-based, human studies (Balfoussia et al., 2014). In a prior study from our research group, endurance athletes served as their own controls and in random, counterbalanced order either exercised intensely for 2.5 h, 3 days in a row, or sat in the lab (Nieman et al., 2018). Fingerprick samples for dried blood spot samples (DBS) were collected from study participants before and after laboratory-based exercise or rest sessions, and then during two recovery days. From this pilot study, thirteen proteins were linked to functional overreaching (FOR), and most were associated with the acute phase response and innate immune system activation.

Thus, the purpose of this study was to use the recently developed targeted panel of proteins in a group of high-level swimmers over the course of the entire season to track physiologic changes that may provide biologic markers of internal and external stress. In order to create a full pictures of these changes over the course of the competitive swim season, various other measures were collected and included in the statistical model: training workload and performance assessment, quantified feedback from coaches, and perceived training distress and illness. We hypothesized that the use of blood sample proteomic data, in combination with the self-reported training distress and illness data, would enhance the ability to identify periods of highest risk of inappropriate load management, and potential overreaching in swimmers.

## Materials and Methods

### Study Participants

Thirty-five swimmers were initially recruited to the study (Sprint Specialty *n* = 14; Middle Distance Specialty *n* = 17; and Distance Specialty *n* = 5). Participants voluntarily signed informed consent forms, and all research procedures were approved by the Queens University of Charlotte Institutional Review Board (FILE #5-17-BCOH-363). Participants completed baseline testing within the first two weeks of recruitment. Demographic and training histories were acquired with questionnaires. Height, body mass, and percent body fat were measured (seca Medical Body Composition Analyzer 514 bioelectrical impedance scale, Hanover, MD, United States). VO2max was assessed using the Bruce’s treadmill protocol, with oxygen consumption and ventilation continuously monitored using the Cosmed Fitmate metabolic system (Cosmed, Rome, Italy) (Nieman et al., 2006). Swimmers in this study were competing for an NCAA Division II school, and both men’s and women’s swim teams won the national title the year of data collection (which was the 4th national title consecutively). The average number of “pool” hours ranged from 15–18 per week (depending mainly on the specialty sub-group of the swimmers, with distance swimmers clocking more pool hours). The number of weight room hours was not quantified in this study.

### Research Design

Athletes provided blood samples, illness symptoms, and ratings of training distress every Monday morning for 19 weeks out of the 25-week season. On Mondays at approximately 5:45 am, the athletes reported to the training facility in their normal state (no restrictions on food or water or rest) and completed the Training Distress Scale (TDS) and Wisconsin Upper Respiratory Symptom Survey (WURSS) questionnaires (Grove et al., 2014; Barrett et al., 2005). Fingerstick blood samples were then collected using the Volumetric Absorptive Microsampling (VAMS) technology (Neoteryx, Torrance, CA, United States). Coaches provided weekly feedback on each athlete regarding swim performance, signs of training distress, and training intensity (10-point Likert scale, with 10 being the most intense). Coaches used these qualifiers when recording swimmer performance: 1-Below expectations, 2-fair, 3-average, 4-better than expected, and 5-far exceeded expectations. For training distress, the coaches used these qualifiers: 1-no signs, 2-slightly distressed, 3- distressed, 4- recognizable fatigue and drop in performance, and 5- likely FOR with inability to train at expected levels. Coaches were sent the list of swimmers participating in the study with the above questions in a spreadsheet that they filled out based on the previous week of observations. The same coach assessed the same swimmers to reduce inter-rater variability.

### Training Distress Scale and Illness Monitoring

The TDS (Grove et al., 2014) includes 19 questions, and the athletes reported symptoms from the previous week using a 0–4 scale. The total TDS score was calculated using the sum of responses from the 19 questions. The first question of the WURSS (Barrett et al., 2005) was used to monitor illness severity from the previous week (0 = not sick, 1 = very mild URTI to 7 = severe).

### Proteomics Profiling

Dried blood spot (DBS) specimens (approximately 1–2 drops of blood, or 40–60 μL of blood/sample) using VAMS (Neoteryx, Torrance, CA, United States) were dried overnight and stored with desiccant. Proteins from the DBS samples were solubilized and reduced in 80 μl 8 M Urea, 50 mM AmBiC and 0.1 mM DTT for 30 min at 37°C. 6 μl of the protein content (∼30 μg) was transferred to a new 96 wells plate and 1pmol heavy standards was added together with 0.6 μg of trypsin (1:50) in a total volume of 45 μl of 50 mM AmBiC. Tryptic digestion was done O/N while shaking at 37°C. The next day 5 μl of 10% FA was added to quench the tryptic digestion. Samples were cleaned up using C18 reverse phase columns in 96 well plate format (Waters Sep-Pak^®^, C18, 40 mg) and dried down. All samples were randomized and subsequently measured consecutively by mass spectrometry (MS). MS analyses were performed on a Triple Quad mass spectrometer (Agilent 6460 Triple Quad) coupled to a normal flow LC autosampler (Agilent 1290 Infinity). 20 μg of peptide of each sample was injected and peptides were separated with reverse phase normal flow LC chromatography. Samples were loaded on a Liquid Chromatography, 2.1 cm × 25 cm C18 2.7 μm 120 Å column (651750-902, AdvanceBio Peptide Mapping, Agilent) with a flow rate of 0.2 mL/min (buffer A, HPLC H_2_O, 0.1% FA; buffer B, 100% ACN, 0.1% FA; 40-min gradient; 0–3 min: 5% buffer B, 3–18 min: 5 to >25% buffer B, 18–26 min: 25 to >29.5% buffer B, 26–30 min: 29.5 to >40% buffer B, 30–35 min: 95% buffer B, 35–40 min, 5% buffer B). Peptides were transferred to the gaseous phase with positive ion electrospray ionization at 5.0 kV. Gas flow was 12 l min^–1^, cell accelerator voltage was set to 4 and the cycle time was set to 1,500 ms to measure 10 data points per peptide peak. Collision energy and fragmentor were peptide specific and optimized prior to measurements. Spectra were analyzed for quality using Skyline (MacCoss Lab Software^1^) with manual validation.

#### Method Development and Limit of Quantitation

In order to reliably identify the targeted peptides, heavy labeled synthetic peptides were ordered for every targeted peptide (JPT Peptide Technologies, Berlin, Germany). Using Skyline the retention time of each peptide was measured and the fragmentation of each peptide was optimized by changing the collision energy for every peptide. The MS method was optimized using Masshunter software (Agilent Technologies, Santa Clara, CA, United States) with the requirement of acquiring at least 10 data points across all peptide peaks for accurate quantitation. Isolation windows were set to 1 min. As a last step, for every peptide a Limit of Quantitation (LOQ) curve was made to ascertain that the quantity of peptides measured was within the linear range of the curve. Heavy standards were spiked in the DBS matrix in 8 different concentrations and every concentration was injected three times as technical replicates. When the coefficient of variation (CV) was greater than 0.2, the data at these intensities for a particular peptide were considered too variable to be used for quantitation purposes and were not used for further analysis.

#### Library Creation

For high throughput data analysis, a library was created based on the fractionation information of heavy labeled standards (JPT peptide technologies) obtained during method development. The library included the information both for native “light” peptides and their “heavy” labeled synthetic counterparts. The format of the library was applicable to the OpenSWATH workflow (Röst et al., 2014) used for Data Independent Acquisition (DIA).

#### Data Processing

The MRM (Multiple Reaction Monitoring) files were processed using OpenSWATH software and Skyline. Skyline was used for method development and peptide verification. OpenSWATH was used for high through-put data MRM signal processing in order to identify and quantify peptides. After the first data processing by OpenSWATH, the data was treated as follows: first, peaks were smoothened followed by intensity correction of the fragments according to the ratio of the fragments as was present in the library (as defined during method optimization). Peptides were then filtered based on their LOQ results; only peptides that had an intensity in the linear range of the LOQ curve were kept for further analysis. Data was then normalized based on the median intensity of the signal of the heavy peptides. Peptides were finally merged into proteins based on their relative median intensity.

### Statistical Analysis

Principal Component Analysis (PCA) was used from the FactoMiner R library to analyze TDS scores to find the subspace corresponding to the maximum-variance directions in the original space (Lê et al., 2008). In order to obtain the meaningful factors which explained the higher variation, 32 linear mixed models were created by combining different variables, with consideration for a random effect intercept by the athlete. The variables included in this analysis were the coaches rating of distress (Coach_distr) and performance (Coach_perf) for the athletes, swimmer group (based on swim race distance), sex, URTI (using binary data, with “0” indicating no illness, and “>0” indicating illness during the defined time period), and the first principal component as a response variable. *P*-values were obtained through the ANOVA procedure.

A Generalized Linear Mixed Models (GLMM) approach was used to model the response of the 12 targeted proteins across the weeks compared to the baseline week. In the models, the athlete was used as the random effect and sex as the fixed effect. Pairwise comparisons between each time-by-condition level were calculated using the glht function from multcomp R package (Hothorn et al., 2008). The Tukey correction for multiple comparisons was applied to adjust the significance level in this study.

The (LDA analysis was used to evaluate the separation between groups based on different time periods: baseline, and when athletes were taking exams, competing in meets, or engaged in both exams and competitive meets. In the beginning, LDA was focused only on meta-data (TDS, and URTI = 0/>0) and then secondly, the analysis focused on the additional effect of the protein data. The LDA analysis included leave-by-one CV and calculated *p*-values from the Kruskal–Wallis non-parametric rank sum test (Kruskal and Wallis, 1952).

The LASSO (Least Absolute Shrinkage and Selection Operation) logistic regression model (Tibshirani, 1996) was used to evaluate differences between time segments (baseline versus exams, meets, and meets and exams, and also meets versus meets and exams). LASSO is a powerful regularization technique and incorporates an L1- penalization term into the loss function forcing some coefficients to be zero. Differences between scores from LASSO output were compared using the Kruskal–Wallis non-parametric rank sum test (Kruskal and Wallis, 1952).

## Results

Of the thirty-five swimmers recruited to the study, 32 swimmers completed the protocol. See Table 1 for subject characteristics.

**TABLE 1:** Characteristics of the *n* = 32 study participants (mean ± SE).

Variable	Male (*n* = 19)	Female (*n* = 13)
Age (years)	19.9 ± 0.4	20.0 ± 0.3
Height (m)	1.83 ± 0.02	1.69 ± 0.02
Weight (kg)	75.6 ± 3.0	64.8 ± 2.3
% Body fat	12.7 ± 1.2	22.6 ± 1.2
VO_2__max_ (ml^.^kg^–1^min^–1^)	55.9 ± 1.4	48.1 ± 1.9
**Second formatting option for Table 1:**		
Age (years)	19.9 ± 0.4	20.0 ± 0.3
Height (m)	1.83 ± 0.02	1.69 ± 0.02
Weight (kg)	75.6 ± 3.0	64.8 ± 2.3
% Body fat	12.7 ± 1.2	22.6 ± 1.2
Total Lean Muscle (Kg)	33.4 ± 3.4	21.6 ± 2.5
VO_2max_ (ml^.^kg^–1^min^–1^)	55.9 ± 1.4	48.1 ± 1.9

**FIGURE 1:**
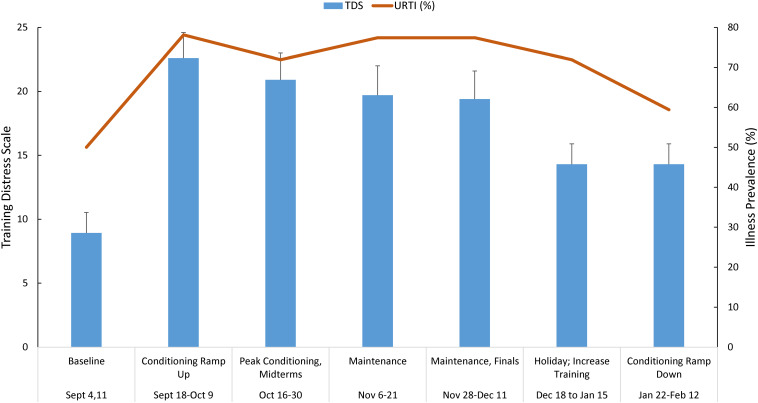
Training distress scale (TDS) total scores (mean ± SE) and upper respiratory tract infection (URTI) prevalence grouped (2–3 weeks running averages) across the 25-week season in *n* = 30 swimmers. TDS and illness significantly increase above baseline, and remained elevated.

Figure 1 shows the TDS scores (mean ± SE) and URTI prevalence across the 25-week season in the swimmers. The data were grouped in 2–3 weeks segments to facilitate interpretation and analysis of the data with respect to training blocks and academic periods. TDS scores varied between athletes, and were highest during the early fall conditioning ramp up period (8.9 ± 1.6 at baseline to a peak of 22.6 ± 2.0). The percent of athletes reporting illness was high throughout the season (50.0–78.1%).

**FIGURE 2:**
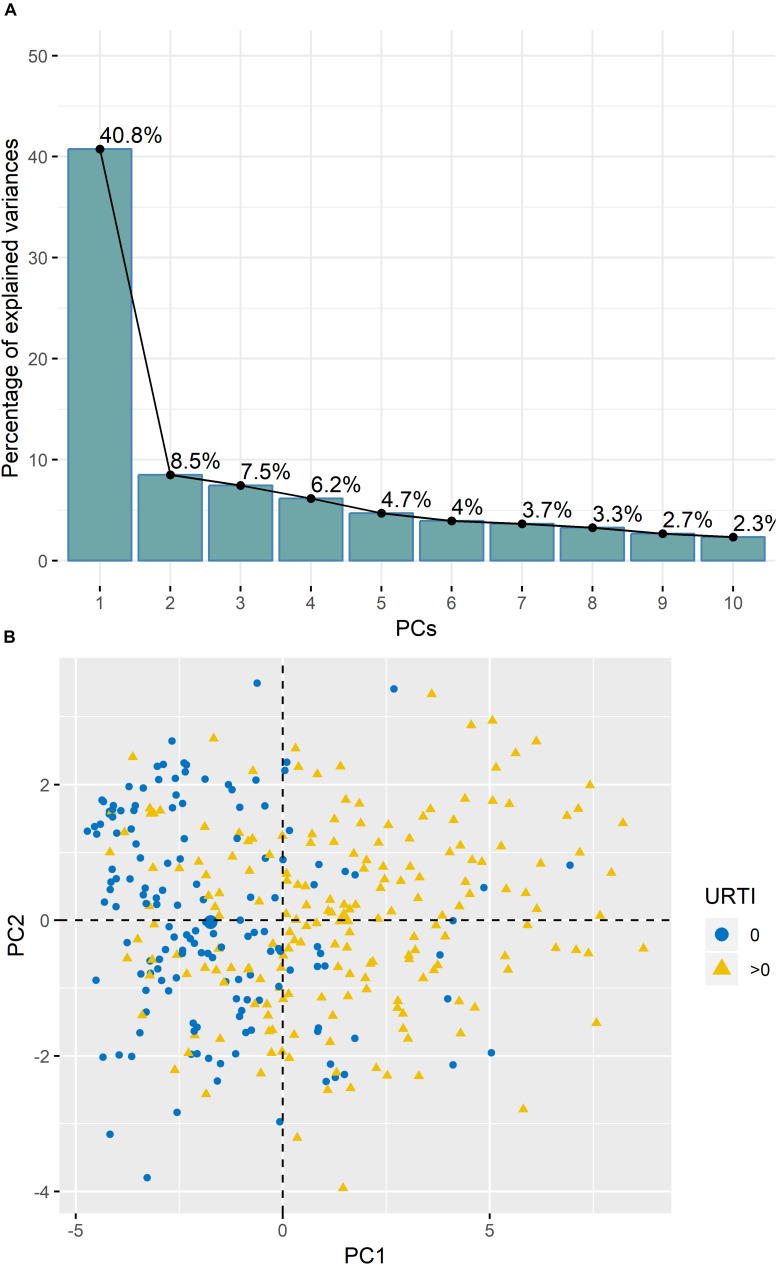
**(A)** Variances of the Principal Components (PC’s) from the PCA analysis of the TDS questionnaire data indicated that ∼40.5% of variance was explained by PC1. **(B)** PC1 was largely attributed to illness prevalence (URTI) (0 = no illness; >0 = illness).

**FIGURE 3:**
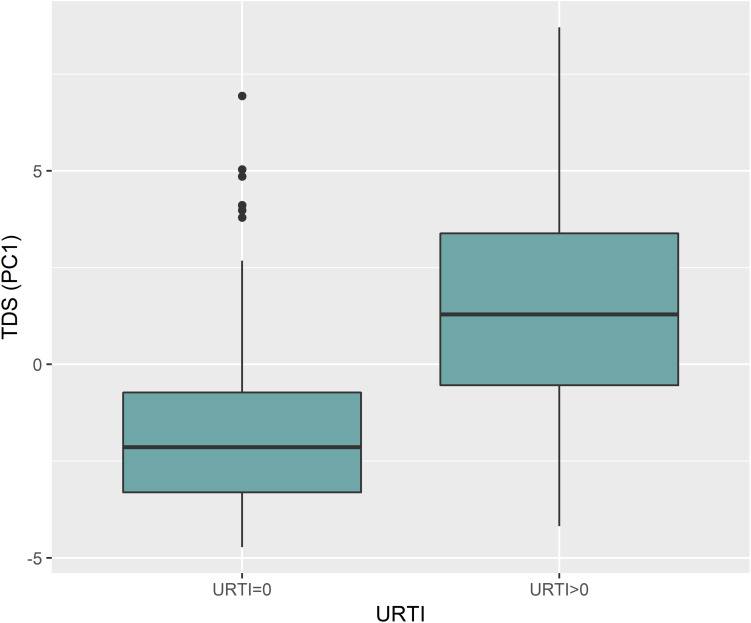
PC1 for TDS was largely attributed to illness prevalence (URTI). The boxplot shows the differences between TDS scores based on URTI prevalence for PC1 (*p* < 0.001); (0 = no illness; >0 = illness).

Analysis of TDS data using PCA revealed that 40.8% of the variance was explained by PC1 (Figure 2A). PC1 was largely attributed to URTI prevalence (Figure 2B). Boxplots in Figure 3 show the differences in TDS scores based on URTI prevalence for PC1. The centered PC1 TDS scores for the athletes reporting illness and no illness were different across the season (*P* < 0.001).

**FIGURE 4:**
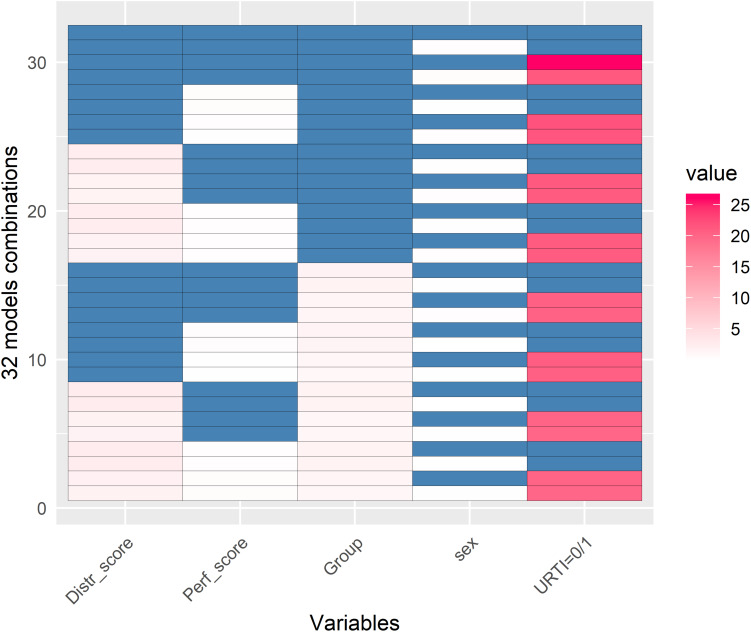
Generalized Linear Mixed Models (GLMM) representation of 32 GLMM models (*y*-axis) with the aim to see which of these 5 variables (*x*-axis) could best explain PC1 variance of the TDS Principal Component Analysis (PCA). Blue and white colors represent no significant relationship with PC1 when a variable was or was not taken into account in the respective model (white and blue, respectively). Red represents when there was a relationship between the variable and PC1 (with darker red representing a stronger association). This figure clearly shows that PC1 was largely attributed to URTI and only to some extent to the Distress score and Group (pink color). Values are in –log(*p*-value). This GLMM analysis shows that (a) there was a strong correlation between PC1 and URTI (0 = no illness, >0 = illness); (b) there was no significant correlations between PC1 and the coaches’ ratings of distress (Distr_score) or swim performance (Perf_score) in the athletes, the swimmer training group (Group = sprinters, middle-, and long-distance), or sex (male and female).

The GLMM representation of 32 analysis models is shown in Figure 4. The goal of this analysis was to explain PC1 variance of the TDS PCA. This analysis showed that there was a strong correlation between URTI and PC1 (red colors). The coaches’ ratings of swim performance and swimmer’s distress, swimmer’s sex, and racing distance were not correlated with PC1.

**FIGURE 5:**
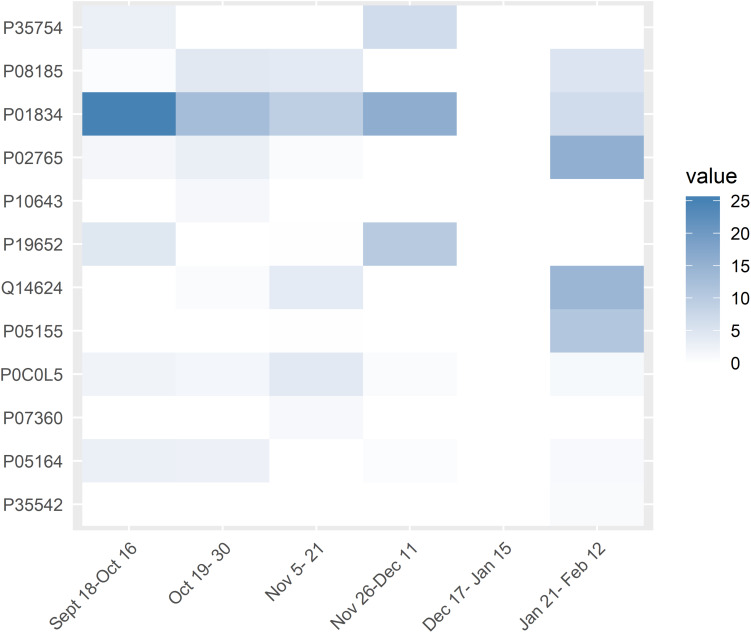
Generalized Linear Mixed Models results of the 12 targeted chronic proteins in the study (*y*-axis) across the weeks (*x*-axis). White rectangles mean that there was no significant difference in expression for the specific protein for that week compared to the baseline weeks. The intensity of blue color of the rectangles indicates the significance of the difference in expression of the proteins between those weeks and the baseline values. Values are in –log(*p*-value).

**FIGURE 6:**
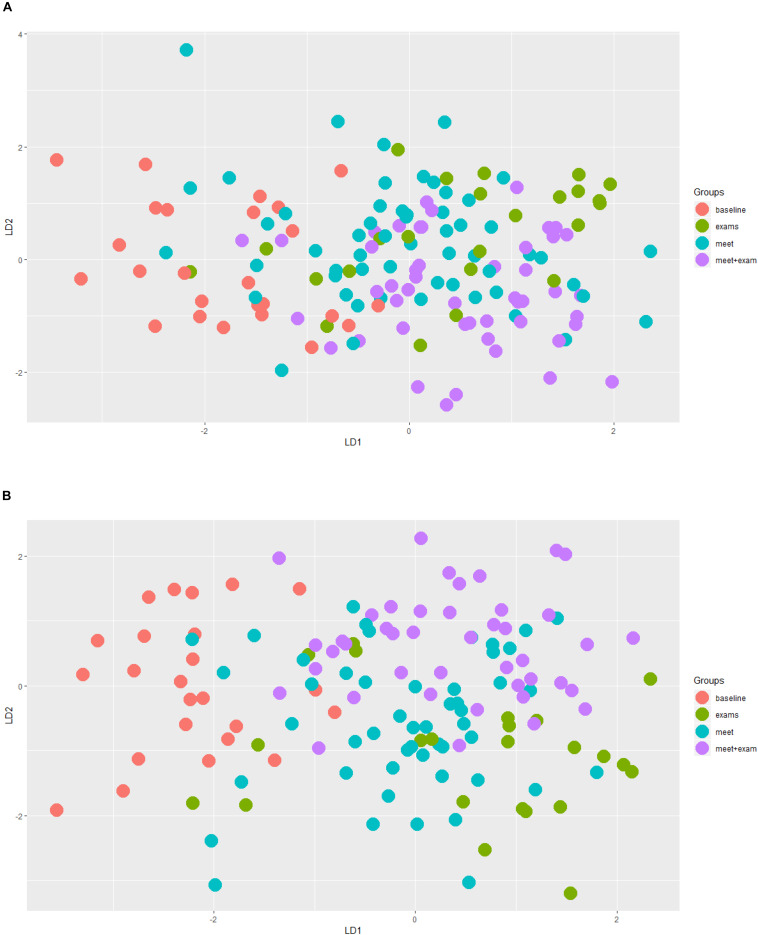
**(A)** Linear Discriminant Analysis (LDA) analysis taking into account TDS, illness, and protein data. Red = baseline weeks; green = exam weeks; blue = swim meet weeks; purple = exam and swim meet weeks. This analysis without the protein expression data supported a separation of baseline weeks from all other weeks (*p* < 0.001, Kruskal–Wallis). **(B)** The same LDA analysis after the inclusion of the 12 selected proteins. This analysis showed an improved, distinct separation of the baseline weeks from all other time periods (*p* < 0.001, Kruskal–Wallis).

**FIGURE 7:**
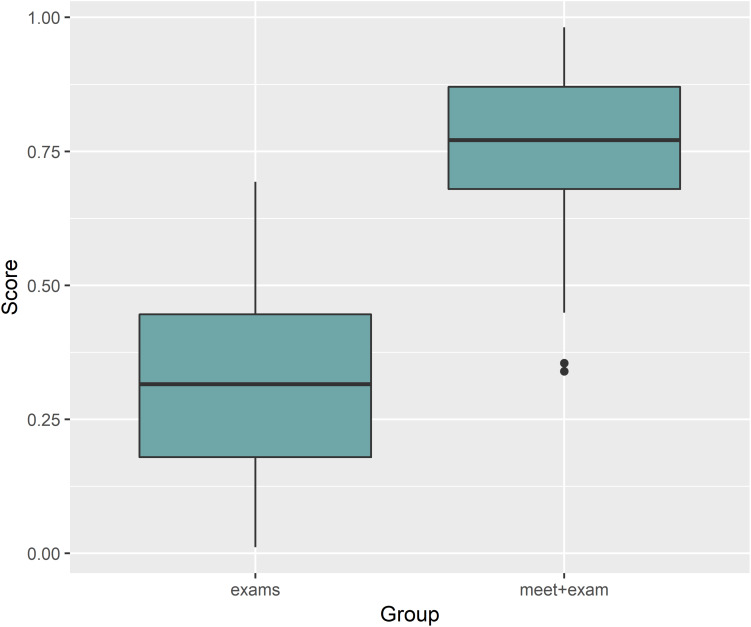
Logistic LASSO regression including the TDS total score, coaches’ ratings of swimmer’s distress and performance, and 12 selected blood proteins showed a significant separation when comparing exams and meets with exams time segments (*p*-value < 0.001). The regression analysis between these time periods was not significant when the protein data were removed.

Generalized Linear Mixed Models analysis of protein expression across the season in the swimmers is shown in Figure 5. The data support protein expression during selected weeks. To improve interpretation of these data, LDA analysis was conducted with TDS and URTI data included and sorted into periods when the swimmers were taking exams with normal training (exams), engaging in competitive meets (meets), and when the swimmers were taking both exams and competing in meets (meet-exam). This LDA analysis was conducted both with and without the proteomics data (Figures 6A,B). This analysis showed a separation of the baseline weeks from weeks with exams, meets, or both exams and meets (*p* < 0.001, Kruskal–Wallis). Adding the panel of protein expression data enhanced the separation between these groups of weeks (*p* < 0.001, Kruskal–Wallis). Figure 7 depicts results from the logistic LASSO regression that included the TDS total score, coaches’ ratings of swimmer’s distress and performance, and 12 selected blood proteins. This analysis showed a separation of weeks when exams were taken from weeks when both competitive swim meets and exams were engaged in (*p*-value < 0.001). The regression analysis between these time periods was not significant when the protein data were removed.

## Discussion

This study tracked changes in a targeted group of proteins, recently identified as potential biomarkers of overreaching in athletes (Nieman et al., 2018), in high level swimmers over the course of the entire season. Indeed, combining proteomic data with self-reported training distress and illness, allowed for identification of periods of high physiologic stress and potential overreaching in swimmers.

The swimmers in this study experienced high levels of distress and URTI (prevalence of 50.0–78.1% during each 2 to 3-week segment) throughout the 25-week season, confirming reports from other investigators (Hellard et al., 2015; Collette et al., 2018). Hellard et al. (2015) followed 28 elite swimmers for 4 years and showed that the odds of URTI were 50–70% higher during intensive training periods. Studies and comprehensive reviews generally support the relationship between training loads and illness, especially during periods of intensification and competition (Fricker et al., 2005; Gleeson et al., 2013; Aubry et al., 2014; Drew and Finch, 2016; Jones et al., 2017).

The physiologic and psychologic response to both internal and external stress is highly individualized in swimmers (Collette et al., 2018). This variance underscores the importance of adding physiologic biomarkers to aid in the identification of overreaching and overtraining. In a previous study conducted by our research group, a targeted panel of proteins was identified that could be utilized and validated in future investigations of overreaching and overtraining (Nieman et al., 2018). These proteins can be analyzed in fingerprick DBS samples, improving the potential practical application of this technology to athletic settings compared to full blood draws. The targeted protein panel met the requirement that changes in response to acute exercise should be distinguishable from chronic changes, and be relatively easy to collect and measure (Meeusen et al., 2013). Our analysis found that most of these targeted proteins were involved in the immune defense response including the acute phase response, complement activation, and humoral responses mediated by circulating immunoglobulins (Nieman et al., 2018). The acute phase response is a systemic reaction to prolonged exercise stress, and involves the production of many proteins including serum amyloid A (SAA), complement and transport proteins, antiproteases, and those involved with the coagulation and fibrinolytic system (Gabay and Kushner, 1999; Balfoussia et al., 2014). Liver production of SAA rises strongly during the acute phase response, and is involved in signaling pathways related to phagocyte migration and inflammation (Ye and Sun, 2015). Although more human studies are needed to improve practical applications in the athletic setting, SAA, one of the proteins in the targeted protein panel (Table 2), has been used in studies of race horses and dogs as an indicator of exercise-induced muscle damage and poor performance (Cywinska et al., 2010, 2013; Casella et al., 2013; Valle et al., 2015).

**TABLE 2:** The 12 targeted proteins measured in this study based on the data from Nieman et al. (2018).

UniProt protein	Protein name	Function
P35542	Serum amyloid A-4 protein	Major acute phase reactant; cell chemotaxis
P05164	Myeloperoxidase	Granulocyte microbicidal activity; production of hypochlorous acid
P07360	Complement component C8 gamma chain	Part of membrane attack complex; forms pores in target cells
P0C0L5	Complement C4B	Non-enzymatic component C3, C5 convertases; complement activation; inflammation
P05155	Plasma protease C1 inhibitor	Crucial role in complement activation.
Q14624	Inter-alpha-trypsin inhibitor heavy chain H4	Acute-phase protein involved in trauma inflammatory response
P19652	Alpha-1-acid glycoprotein 2	Transport protein; modulates immune; acute-phase; inflammation
P10643	Complement component C7	Part of membrane attack complex; forms pores in target cells
P02765	Alpha-2-HS-glycoprotein	Promotes endocytosis; acute-phase response; phagocytosis; bone mineral
P01834	Ig kappa chain C region	Antigen and Ig receptor binding; complement activation; innate; phagocytosis
P08185	Corticosteroid-binding globulin	Major transport protein for glucocorticoids and progestins
P35754	Glutaredoxin-1	Glutathione activity; cell redox homeostasis

The data of the current study supports the use of this targeted protein panel in combination with ratings of training distress when monitoring athletes during a competitive training season. The chief limitation of the current study was the lack of a suitable control group, and future research will help define DBS protein levels that when combined with TDS scores predict overtraining. TDS, illness, and proteomics data can be used to support decisions by the coaches or performance monitoring team regarding alleviation of both internal and external loads for the athlete. A unique finding in this study was the apparent importance of the internal stressors to the University level athlete, such as academic exam periods, psychological pressure to perform athletically, and other personal stressors. These internal stressors contribute to the physiologic response in the body to external training loads, and future research should investigate strategies to mitigate the negative response of both internal and external stressors of athletes.

## Conclusion

This study showed that training distress was strongly related to URTI prevalence in collegiate swimmers, and that proteomics data added strength to this relationship, especially during high stress periods when the athletes were involved with exams and/or competitive swim meets. Coaches feedback regarding training distress and level of swim performance did not correlate well with the swimmer’s TDS scores, confirming the findings of other studies (Brink et al., 2014, 2017). One potential cause for the disconnect between athlete and coach is that periods of physiologic stress are related at least in part to factors outside the training program. According to the NCAA, 73% of college athletes believe their coach cares about their overall well-being (Paskus, 2016). Despite this, 30% of athletes report they have been overwhelmed during the past month, and desire more focus by the coaches on their education, sleep, and nutrition. Division II NCAA athletes report spending 32.5 h/week on athletics, and up to 38.5 h/week on academics (Paskus, 2016). Coaches often do not account for all of the stresses experience by their athletes, including academic, social, financial, and living challenges. Further research using proteomics data as an objective measure will refine strategies to reduce training stress and illness prevalence among elite athletes, and improve coach’s perceptions of training-related distress.

## Data Availability Statement

The datasets generated for this study are available on request to the corresponding author.

## Ethics Statement

The studies involving human participants were reviewed and approved by Institutional Review Board, Queens University of Charlotte. The patients/participants provided their written informed consent to participate in this study.

## Author Contributions

AK, DN, LZ, AG, and AP designed the study and drafted the manuscript. AK collected the data. AG and AP analyzed the blood samples. AK, DN, LZ, AG, and AP analyzed the data. All authors are responsible for revising the intellectual content of the manuscript, and reading and approving the final version of the manuscript.

## Conflict of Interest

AG and AP are both founders of the company ProteiQ Biosciences GmbH in Berlin, Germany. LZ was under contract with ProteiQ Biosciences GmbH at the time of statistical analysis for this paper. The remaining authors declare that the research was conducted in the absence of any commercial or financial relationships that could be construed as a potential conflict of interest.

## Footnotes

1 https://skyline.ms/
